# Usefulness of scoring right ventricular function for assessment of prognostic factors in patients with chronic thromboembolic pulmonary hypertension

**DOI:** 10.1007/s00380-018-1168-7

**Published:** 2018-04-27

**Authors:** Yoshihiro Kamimura, Naoki Okumura, Shiro Adachi, Shigetake Shimokata, Fumitaka Tajima, Yoshihisa Nakano, Akihiro Hirashiki, Toyoaki Murohara, Takahisa Kondo

**Affiliations:** 1 Nagoya University Graduate School of Medicine, 65 Tsurumai-cho, Shouwa-ku, Nagoya, 466-8550 Japan; 2 Department of Advanced Medicine in Cardiopulmonary Disease, Nagoya University Graduate School of Medicine, 65 Tsurumai-cho, Shouwa-ku, Nagoya, 466-8560 Japan; 3 Department of Cardiology, National Center for Geriatrics and Gerontology, Morioka-cho 7-430, Obu, 474-8511 Japan

**Keywords:** Chronic thromboembolic pulmonary hypertension, Echocardiography, Right ventricular function

## Abstract

Right ventricular (RV) function is associated with prognosis in chronic thromboembolic pulmonary hypertension (CTEPH). This study aimed to establish an RV dysfunction score using RV echocardiographic parameters to clarify the clinical characteristics in patients with CTEPH and to compare RV dysfunction score with parameters such as World Health Organization (WHO) functional class, hemodynamics, exercise capacity, and plasma BNP level. We enrolled 35 inpatients with CTEPH (mean age, 62 ± 15 years, 15 males). We constructed ‘an RV dysfunction score’ calculated as the summation of each point awarded for the presence of four parameters: tricuspid annular plane systolic excursion (TAPSE) < 16 mm, 1 point; tissue Doppler-derived tricuspid lateral annular systolic velocity (*S*′) < 10 cm/s, 1 point; right ventricular fractional area change (RVFAC) < 35%, 1 point; and right ventricular myocardial performance index (RV-MPI) > 0.4, 1 point. TAPSE, *S*′, RVFAC, and RV-MPI was 18.7 ± 4.8 mm, 11.9 ± 3.1 cm/s, 33.5 ± 13.9%, and 0.39 ± 0.2, respectively. The RV dysfunction score was associated with symptom [WHO functional class (*p* = 0.026)], hemodynamics [mean PAP (*p* = 0.01), cardiac index (*p* = 0.009), pulmonary vascular resistance (*p* = 0.001), and SvO_2_ (*p* = 0.039)], exercise capacity [6-min walk distance (*p* = 0.046), peakVO_2_ (*p* = 0.016), and VE/VCO_2_ slope (*p* = 0.031)], and plasma BNP level (*p* = 0.005). This RV dysfunction score using the four RV echocardiographic parameters could be a simple and useful scoring system to evaluate prognostic factors in patients with CTEPH.

**Electronic supplementary material:**

The online version of this article (10.1007/s00380-018-1168-7) contains supplementary material, which is available to authorized users.

## Introduction

Chronic thromboembolic pulmonary hypertension (CTEPH) continues to be a disease with poor prognosis, although several specific drugs and treatment are available [1, 2]. Echocardiography is widely used for assessing the severity of pulmonary hypertension and right ventricular (RV) function, which is important for prognosis in patients with CTEPH. The American Society of Echocardiography (ASE) recommends four RV echocardiographic parameters for the assessment of RV function: tricuspid annular plane systolic excursion (TAPSE), tissue Doppler-derived tricuspid lateral annular systolic velocity (*S*′), right ventricular fractional area change (RVFAC), and right ventricular myocardial performance index (RV-MPI) [3–7]. However, there is still discussion regarding which echocardiographic parameter is best associated with RV function, and also is capable of predicting outcome. RV has a unique and complicated contraction pattern: [8–10] Hence, RV function needs to be assessed comprehensively. We hypothesized that RV function could be evaluated more precisely if the four RV echocardiographic parameters were combined.

This study aimed to establish an RV dysfunction score using the four RV echocardiographic parameters (TAPSE, *S*′, RVFAC, and RV-MPI) to clarify the clinical characteristics on admission in patients with CTEPH and to compare the RV dysfunction score with parameters such as symptoms of World Health Organization (WHO) functional class, hemodynamics, exercise capacity [6-min walk test (6MWT) and cardiopulmonary exercise test (CPET)], and plasma brain natriuretic peptide (BNP) level used for risk assessment in patents with CTEPH.

## Materials and methods

### Study individuals

We enrolled 35 consecutive patients with CTEPH admitted to our institution between April 1, 2015 and Aug 31, 2017. CTEPH was diagnosed as a mean pulmonary arterial pressure (PAP) of ≥ 25 mmHg and a pulmonary arterial wedge pressure (PAWP) of < 15 mmHg by right heart catheterization (RHC), ventilation-perfusion lung scintigraphy, computerized tomography, and/or pulmonary angiography after at least 3 months anticoagulant treatment. Pregnancy, hypersensitivities to the contrast medium, and renal dysfunction were excluded. This study was approved by the human research ethics committees of Nagoya University Hospital (no. 2014-0332), and all patients gave written informed consent.

### Doppler echocardiography

Two-dimensional, M-mode, and Doppler echocardiographic images were acquired (iE33; Philips Healthcare, Eindhoven, the Netherlands) and examined in accordance with the ASE guidelines [6]. Patients were examined in the left decubitus position through parasternal long-axis, short-axis, and apical views. TAPSE was measured with M-mode imaging as the distance of systolic excursion of the lateral tricuspid valve annular segment along its longitudinal plane from the RV-focused apical 4-chamber window. Tissue Doppler echocardiography was performed in the RV-focused apical four-chamber view, with the tissue sampling volume located at the lateral side of the tricuspid annulus, and the *S*′ was measured. The percentage RVFAC was defined as (end diastolic area – end systolic area)/end diastolic area × 100. RV-MPI was defined as the ratio of isovolumic time divided by ejection time.

### Hemodynamic studies

All patients underwent RHC via the right internal jugular vein with a 6-French Thermodilution catheter (Goodman Co. Ltd., Nagoya, Japan) to obtain the PAP, PAWP, RV pressure (RVP), and right atrial pressure (RAP). Mixed venous oxygen saturation (SvO_2_) and arterial oxygen saturation (SaO_2_) were measured in blood drawn from the main pulmonary artery and radial artery, respectively. Cardiac output (CO) was calculated by using the Fick method, and pulmonary vascular resistance (PVR) was calculated by using the standard formula: PVR = (mean PAP − mean PAWP)/CO. Cardiac index (CI) was calculated by using the formula: CI = CO/body surface area.

### Six-minute walk test

The measurement of 6-min walk distance (6MWD) was performed in all but one patients enrolled in this study. Each patient was instructed to walk at their own pace. The physiotherapist supervised the test, telling the patient the elapsed time every 1 min. Although the patient was allowed to stop and take a rest freely, all patients continued to walk during the test. No patients were terminated prematurely by the test administrator, and no complications occurred. Dyspnea during the test was checked with the modified Borg dyspnea score. Before and during the 6MWT, the peripheral capillary oxygen saturation (SpO_2_) was monitored by saturation monitoring for the safety.

### Cardiopulmonary exercise testing

Cardiopulmonary exercise testing (CPET) was performed in all patients in an erect position on an electronically braked cycle ergometer with breath-by-breath measurements by using an Ergospirometry Oxycon Pro (Carefusion Germany, 234, GmbH, Hochberg, Germany). The exercise protocol consisted of 3 min of rest and 3 min of unloaded cycling, followed by a 10-W/min ramp-incremental protocol. Parameters including oxygen consumption (VO_2_), carbon dioxide output (VCO_2_), and the minute ventilation (VE) were continuously measured by a fixed cardio-pulmonary exercise system through a tightly fitted facemask. The CPET was safely performed without any problem such as syncope, arrhythmia, or worsening of right heart failure.

### Right ventricular dysfunction score

The RV dysfunction score was calculated as the summation of points awarded for the presence of four parameters (TAPSE < 16 mm, 1 point; *S*′ < 10 cm/s, 1 point; RVFAC < 35%, 1 point; and RV-MPI > 0.4, 1 point) using the cut-off value recommended by ASE guidelines [6]. Total scores range from 0 to 4. Patients were then divided into four groups based on their score: score 0 (*n* = 6), score 1 (*n* = 13), score 2 (*n* = 11), and score 3/4 (*n* = 5). Higher score indicates worse RV function.

### Statistical analysis

All analyses were performed using Stata version 14 (Stata Corp., College Station, Texas, USA). Baseline characteristics were compared using the Kruskal–Wallis test for continuous variables and the *χ*^2^ test for categorical variables. Hemodynamics, exercise capacity, and plasma BNP level between the four RV function parameters were compared using the Wilcoxon rank sum test. Trend test was performed among the four RV dysfunction score groups. All reported *p* values were two-sided, and *p* < 0.05 was considered to be statistically significant.

## Results

Table 1 shows the baseline patients’ characteristics according to the RV dysfunction score. Overall, the mean age of all patients was 62 ± 15 years, and 15 (43%) were male. Of these 35 patients, 21 (60%) were receiving oral CTEPH-specific drug therapy, riociguat, prescribed at our hospital. The mean PAP, PVR, CI, and RAP of all 35 patients were 37.2 ± 10.6 mmHg, 8.2 ± 4.7 Wood Unit, 2.3 ± 0.7 l/min/m^2^, and 6.0 ± 3.0 mmHg, respectively. All patients were prescribed anticoagulation, such as warfarin or direct oral anticoagulants. There were no statistically significant differences between four groups in the laboratory findings and pericardial effusion.

**Table 1:** Patients’ characteristics according to the RV dysfunction score

	Overall	RV dysfunction score				*p* value
		0	1	2	3/4	
	(*n* = 35)	(*n* = 6)	(*n* = 13)	(*n* = 11)	(*n* = 5)	
Age (years)	62.0 ± 14.7	59.7 ± 13.0	64.5 ± 14.3	60.1 ± 17.0	62.6 ± 15.9	0.761
Male	15 (42.9%)	2 (33.3%)	5 (38.5%)	5 (45.5%)	3 (60.0%)	0.812
BMI (kg/m^2^)	24.6 ± 5.9	24.0 ± 4.2	25.3 ± 4.3	23.6 ± 4.7	23.0 ± 2.3	0.422
SBP (mmHg)	118.9 ± 23.5	120.4 ± 33.1	107.5 ± 14.0	116.1 ± 14.6	116.3 ± 13.9	0.429
DBP (mmHg)	67.7 ± 12.0	64.1 ± 14.6	64.3 ± 10.7	74.1 ± 13.2	71.0 ± 1.7	0.333
Pulse (/min)	75.1 ± 9.8	76.9 ± 13.3	77.6 ± 8.7	79.6 ± 9.0	78.0 ± 7.0	0.968
SpO_2_ (%)	92.8 ± 4.4	92.7 ± 8.4	95.0 ± 2.9	94.8 ± 2.3	92.3 ± 5.7	0.167
Patients’ history						
Hypertension	10 (28.6%)	1 (16.7%)	4 (30.8%)	4 (36.4%)	1 (20.0%)	0.812
Dyslipidemia	5 (14.3%)	0 (0%)	2 (15.4%)	2 (18.2%)	1 (20.0%)	0.733
Diabetes mellitus	3 (8.6%)	0 (0%)	0 (0%)	3 (27.3%)	0 (0%)	0.67
Acute PE	8 (22.9%)	1 (16.7%)	5 (38.5%)	2 (18.2%)	0 (0%)	0.315
Laboratory data						
Hb (g/dl)	13.8 ± 2.3	12.7 ± 2.6	13.7 ± 2.0	13.9 ± 2.3	15.2 ± 2.6	0.407
AST (IU/l)	23.4 ± 8.9	19.2 ± 3.5	25.7 ± 10.9	22.7 ± 8.6	23.8 ± 9.0	0.625
ALT (IU/l)	20.1 ± 10.9	15.0 ± 5.1	23.9 ± 14.4	20.2 ± 8.0	15.8 ± 9.5	0.332
LDH (IU/l)	215.1 ± 44.2	178.3 ± 33.8	229.4 ± 46.1	216.0 ± 29.4	220.4 ± 62.4	0.13
γGTP (IU/l)	44.7 ± 38.5	23.0 ± 9.4	48.5 ± 27.8	54.5 ± 60.0	39.2 ± 10.7	0.198
HbA1c (%)	6.0 ± 0.6	5.9 ± 0.2	5.9 ± 0.6	6.2 ± 0.9	6.1 ± 0.5	0.564
eGFR (ml/min/1.73 m^2^)	67.8 ± 16.4	70.4 ± 14.7	65.7 ± 14.4	68.6 ± 21.7	68.6 ± 13.8	0.946
BNP (pg/ml)	127.2 ± 184.9	33.1 ± 35.5	46.5 ± 56.2	174.9 ± 253.4	345.0 ± 138.0	0.02
Hemodynamics						
Mean PAP (mmHg)	37.2 ± 10.6	28.8 ± 7.0	35.3 ± 8.2	42.1 ± 12.9	41.6 ± 7.8	0.062
PVR (Wood Unit)	8.2 ± 4.7	4.4 ± 1.4	6.8 ± 2.4	10.1 ± 6.0	12.1 ± 4.8	0.010
CI (l/min/m^2^)	2.3 ± 0.7	2.8 ± 1.0	2.4 ± 0.5	2.3 ± 0.7	1.7 ± 0.2	0.036
RAP (mmHg)	6.0 ± 3.0	5.0 ± 2.5	5.8 ± 3.2	5.7 ± 3.1	8.2 ± 2.2	0.283
SvO_2_ (%)	62.9 ± 8.8	66.2 ± 6.6	66.5 ± 3.8	60.7 ± 10.2	54.5 ± 12.0	0.039
Medication						
DOAC	15 (42.9%)	1 (16.7%)	5 (38.5%)	6 (54.5%)	3 (60.0%)	0.392
Warfarin	20 (57.1%)	5 (83.3%)	8 (61.5%)	5 (45.5%)	2 (40.0%)	0.392
Riociguat	21 (60.0%)	6 (100.0%)	5 (38.5%)	8 (72.7%)	2 (40.0%)	0.044
RV echo parameters						
TAPSE (mm)	18.7 ± 4.8	20.7 ± 1.7	19.7 ± 4.6	18.2 ± 6.3	14.9 ± 2.2	0.093
*S*′ (cm/s)	11.9 ± 3.1	13.6 ± 3.1	12.8 ± 1.7	11.1 ± 4.1	9.5 ± 0.9	0.018
RVFAC (%)	33.5 ± 13.9	42.3 ± 3.9	36.9 ± 9.1	31.4 ± 19.0	18.6 ± 3.8	0.002
RV-MPI	0.4 ± 0.16	0.26 ± 0.1	0.36 ± 0.1	0.4 ± 0.1	0.64 ± 0.2	0.003
Pericardial effusion	10 (28.6%)	1 (16.7%)	4 (30.8%)	3 (27.3%)	2 (40.0%)	0.855

Data are presented as mean ± SD or *n* (%)

*BMI* body mass index, *SBP* systolic blood pressure, *DBP* diastolic blood pressure, *SpO*_*2*_ oxygen saturation, *Acute PE* acute pulmonary embolism, *Hb* hemoglobin, *AST* aspartate aminotransferase, *ALT* alanine aminotransferase, *LDH* lactate dehydrogenase, *γGTP* γ-glutamyl transferase, *eGFR* estimated glomerular filtrating ratio, *BNP* brain natriuretic peptide, *mean PAP* mean pulmonary artery pressure, *CI* cardiac index, *PVR* pulmonary vascular resistance, *RAP* right atrial pressure, SvO_2_ mixed venous oxygen saturation, *DOAC* direct oral anticoagulant. *TAPSE* tricuspid annular plane systolic excursion, *S′* tissue Doppler-derived tricuspid lateral annular systolic velocity, *RVFAC* right ventricular fractional area change, *RV-MPI* right ventricular myocardial performance index

**Fig. 1:**
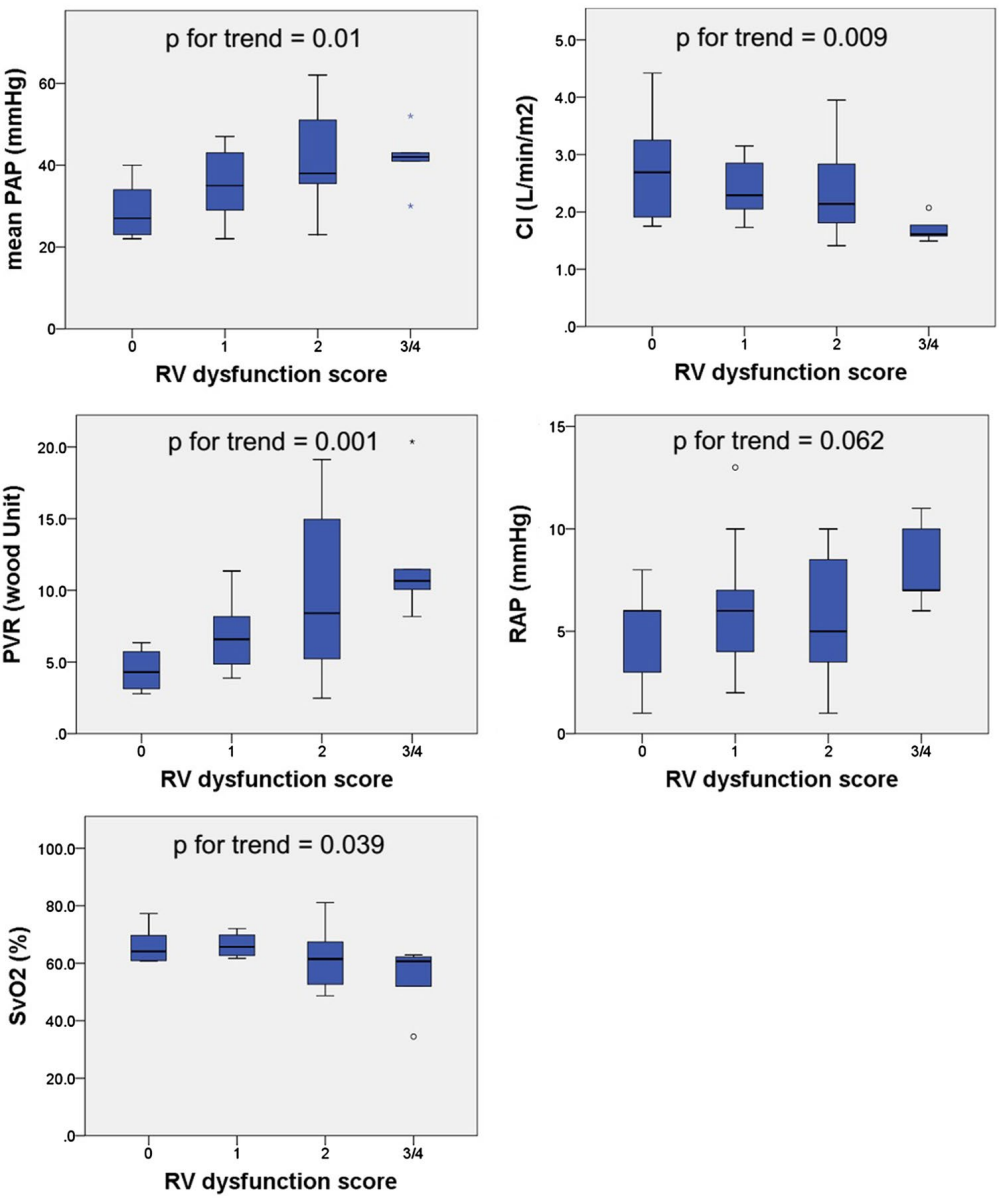
A comparison of the hemodynamics [mean pulmonary artery pressure (PAP), cardiac index (CI), pulmonary vascular resistance (PVR), right atrial pressure (RAP), and mixed venous oxygen saturation (SvO_2_)] according to the RV dysfunction score. The mean PAP, CI, PVR, and SvO_2_ were significantly worsening as the RV dysfunction score increased (*p* = 0.01, *p* = 0.009, *p* = 0.001, *p* = 0.039, respectively). The right atrial pressure showed a considerable trend toward significance (*p* = 0.062)

Figure 1 shows a comparison of the hemodynamics (mean PAP, CI, PVR, RAP, and SvO_2_) according to the RV dysfunction score. The mean PAP, CI, PVR, and SvO_2_ were significantly worsening as the RV dysfunction score increased (p for trend = 0.01, *p* for trend = 0.009, *p* for trend = 0.001, and *p* for trend = 0.039, respectively). The RAP showed a considerable trend toward significance (*p* = 0.062).

**Fig. 2:**
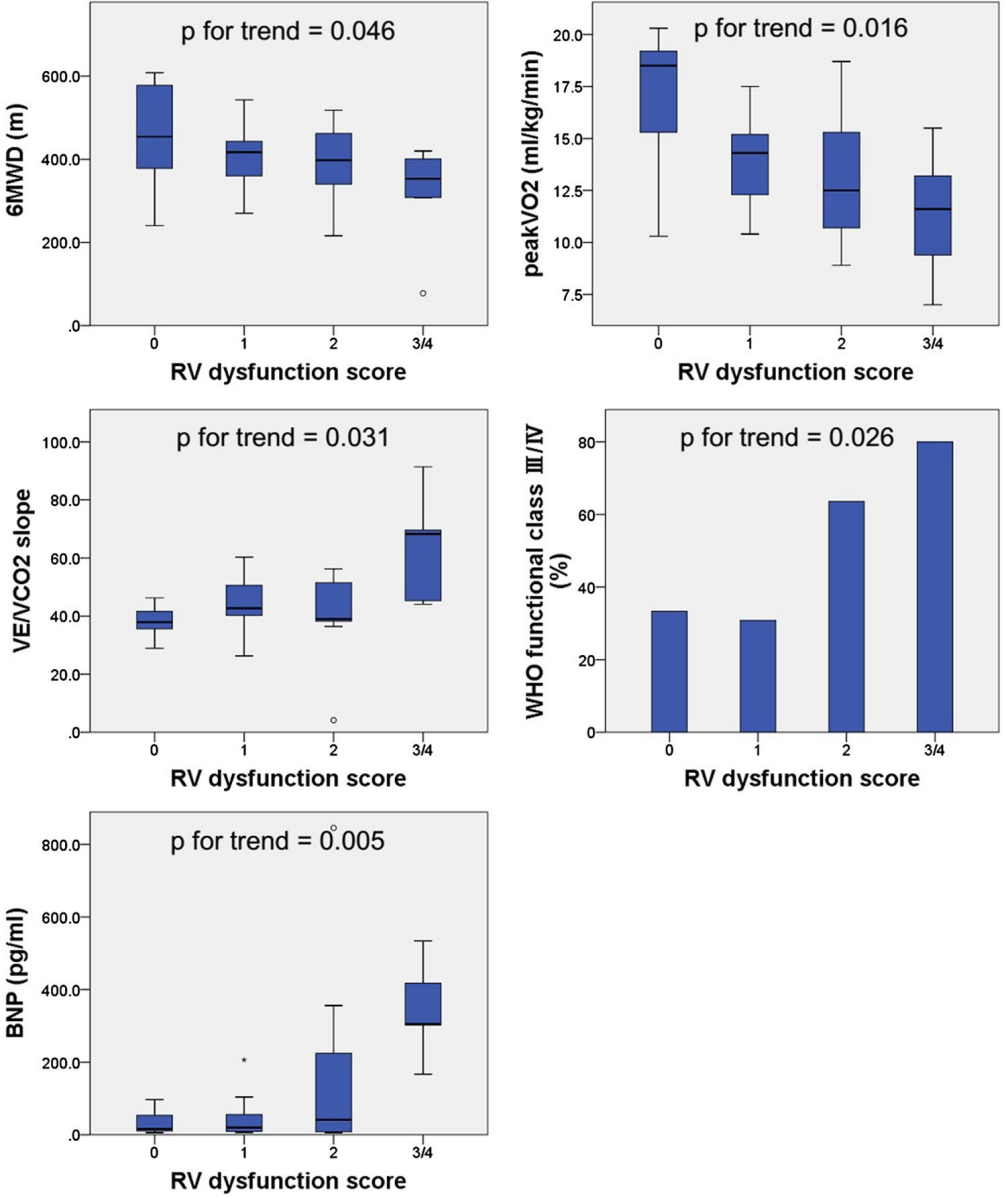
A comparison of the exercise capacity [6-min walk distance (6MWD), and peakVO_2_, VE/VCO_2_ slope)], symptom (WHO functional class), and plasma BNP level according to RV dysfunction score. All parameters showed significant deteriorating trend as the RV function score increased (*p* = 0.046, *p* = 0.016, *p* = 0.031, *p* = 0.026, and *p* = 0.005, respectively)

Figure 2 shows a comparison of the exercise capacity (6MWD, peakVO_2_, and VE/VCO_2_ slope), symptom (WHO functional class), and plasma BNP level according to RV dysfunction score. All parameters showed significant deteriorating trend as the RV function score increased (*p* for trend = 0.046, *p* for trend = 0.016, *p* for trend = 0.026, and *p* for trend = 0.005, respectively).

Table 2 shows the comparison of the baseline hemodynamics, exercise capacity, and plasma BNP levels between the four echocardiographic parameters: TAPSE (≥ 16 vs. < 16), *S*′ (≥ 10 vs. < 10), RVFAC (≥ 35 vs. < 35), and RV-MPI (≤ 0.4 vs. > 0.4). Of these parameters, the correlations between RV-MPI/RVFAC and hemodynamics was stronger than those between TAPSE/*S*′ and hemodynamics. Especially, RVFAC (≥ 35 vs. < 35) showed the strongest correlation with hemodynamics (mean PAP, 32.7 ± 8.6 vs. 41.5 ± 10.7 mmHg, *p* = 0.012; PVR, 5.8 ± 2.5 vs. 10.5 ± 5.3 Wood Unit, *p* = 0.002; CI, 2.7 ± 0.7 vs. 2.0 ± 0.5 l/min/m^2^, *p* = 0.003; RAP, 5.1 ± 2.6 vs. 6.9 ± 3.1 mmHg, *p* = 0.068; and SvO_2_, 67.3 ± 6.0 vs. 58.8 ± 9.2%, *p* = 0.003, respectively). RVFAC also tends to show stronger correlation with exercise capacity and laboratory findings than the other parameters. Since there is a possibility that riociguat positively affected RV function and hemodynamics, sub-group analysis was conducted between the patients with riociguat and without riociguat (Supplementary Table 1). The result showed no significant difference between these two groups. Therefore, we concluded that treating two groups of patients collectively was appraisable. Table 3 shows the correlations among the four RV function echo parameters. Of these four parameters, there were significant correlations between TAPSE and *S*′, and between RVFAC and RV-MPI (*r* = 0.603, *p* < 0.001, and *r* = − 0.461, *p* = 0.005, respectively).

**Table 2:** Hemodynamics, biomarker, and exercise capacity stratified by each echocardiographic parameter

	TAPSE			*S*′			RVFAC			RV-MPI		
	≥ 16 mm	< 16 mm	*p* value	≥ 10 cm/s	< 10 cm/s	*p* value	≥ 35%	< 35%	*p* value	≤ 0.4	> 0.4	*p* value
	(*n* = 26)	(*n* = 9)		(*n* = 26)	(*n* = 9)		(*n* = 17)	(*n* = 18)		(*n* = 18)	(*n* = 17)	
Hemodynamics												
Mean PAP (mmHg)	38.1 ± 10.7	34.8 ± 10.4	0.428	35.8 ± 11.2	41.3 ± 7.7	0.18	32.7 ± 8.6	41.5 ± 10.7	0.012	32.7 ± 8.5	42.0 ± 10.7	0.007
PVR (Wood Unit)	7.9 ± 4.6	8.9 ± 5.3	0.588	7.4 ± 4.5	10.4 ± 4.9	0.097	5.8 ± 2.5	10.5 ± 5.3	0.002	6.4 ± 3.2	10.1 ± 5.4	0.022
CI (L/min/m^2^)	2.3 ± 0.7	2.43 ± 0.9	0.931	2.4 ± 0.7	2.1 ± 0.8	0.315	2.7 ± 0.7	2.0 ± 0.5	0.003	2.6 ± 0.8	2.1 ± 0.5	0.053
RAP (mmHg)	6.2 ± 3.1	5.4 ± 2.6	0.523	5.9 ± 2.9	6.2 ± 3.2	0.799	5.1 ± 2.6	6.9 ± 3.1	0.068	5.1 ± 2.9	7.0 ± 2.8	0.051
SvO_2_ (%)	63.5 ± 7.2	61.2 ± 12.8	0.499	63.3 ± 9.0	61.7 ± 8.7	0.639	67.3 ± 6.0	58.8 ± 9.2	0.003	65.1 ± 6.9	60.5 ± 10.2	0.125
Laboratory data												
BNP (pg/ml)	108.5 ± 176.4	181.4 ± 209.0	0.315	89.5 ± 171.5	236.2 ± 188.2	0.038	30.5 ± 31.6	218.6 ± 222.0	0.002	70.0 ± 102.6	187.8 ± 232.2	0.068
Exercise capacity												
6MWD (m)	416.3 ± 100.5	335.5 ± 127.0	0.071	401.9 ± 118.0	382.4 ± 88.7	0.67	417.9 ± 105.3	379.0 ± 115.3	0.314	421.4 ± 111.0	373.2 ± 108.5	0.21
PeakVO_2_ (ml/kg/min)	14.2 ± 3.1	12.9 ± 3.8	0.322	14.2 ± 3.3	12.8 ± 3.2	0.289	15.2 ± 3.3	12.6 ± 2.9	0.022	14.9 ± 3.5	12.8 ± 2.8	0.066
VE/VCO_2_ slope	43.7 ± 8.3	49.9 ± 24.9	0.479	42.0 ± 11.3	54.8 ± 18.2	0.018	40.7 ± 7.4	49.6 ± 17.8	0.065	41.0 ± 12.1	49.8 ± 15.4	0.067

Data are presented as mean ± SD

*TAPSE* tricuspid annular plane systolic excursion, *S′* tissue Doppler-derived tricuspid lateral annular systolic velocity, *RVFAC* right ventricular fractional area change, *RV-MPI* right ventricular myocardial performance index, *mean PAP* mean pulmonary artery pressure, *PVR* pulmonary vascular resistance, *CI* cardiac index, *RAP* right atrial pressure*, SvO*_*2*_ mixed venous oxygen saturation, *6MWD* 6 min walk distance, *peakVO*_*2*_ maximal oxygen consumption, *VE/VCO*_*2*_ minute ventilation/carbon dioxide production, BNP brain natriuretic peptide

**Table 3:** Correlation matrix for four RV echocardiographic parameters

	TAPSE	*S*′	RVFAC	RV-MPI
TAPSE	*r* = 1			
*S*′	*r* = 0.603	*r* = 1		
	*p* < 0.001			
RVFAC	*r* = − 0.226	*r* = − 0.108	*r* = 1	
	*p* = 0.192	*p* = 0.538		
RV-MPI	*r* = − 0.097	*r* = − 0.035	*r* = − 0.461	*r* = 1
	*p* = 0.579	*p* = 0.841	*p* = 0.005	

*TAPSE* tricuspid annular plane systolic excursion, *S′* tissue Doppler-derived tricuspid lateral annular systolic velocity, *RVFAC* right ventricular fractional area change, *RV-MPI* right ventricular myocardial performance index

## Discussion

In the present study, we demonstrated that our simple RV dysfunction score using four important RV echocardiographic parameters (TAPSE < 16 mm, *S*′ < 10 cm/s, RVFAC < 35%, and RV-MPI > 0.4) was useful for risk assessment in CTEPH patients in terms of symptoms (WHO functional class), hemodynamics, exercise capacity (6MWT and CPET) and plasma BNP level. Patients with a higher RV dysfunction score had higher BNP levels, more impaired exercise capacity, and worse hemodynamics.

RV function is the most important determinant of prognosis in patients with pulmonary hypertension including CTEPH [11–13]. Cardiac magnetic resonance imaging (MRI) and multi detective computed tomography can be used to assess RV function such as RV volumes, mass, and thickness, and provides accurate and reproducible measurements of RV function. Especially, cardiac MRI is currently regarded as the “gold standard” for RV function assessment [14–16]. Although MRI has the advantage of enabling functional cardiac assessment without the need for contrast media injection or exposing patients to radiation, the main drawbacks include high expenses involved, long examination times, problems of claustrophobia, and limited use for patients with device implantations. In contrast, echocardiography remains to be the first-line examination modality for assessing RV function because of less cost, easy availability, and repeatability [17, 18] Additionally, several studies reported that there was a good association between echocardiographic parameters and hemodynamics or RV function assessed by MRI [19–21], suggesting the usefulness of echocardiography.

RV morphology is complicated, and accurate volumetric assessment with two-dimensional echocardiography is difficult. Therefore, we should be careful in assessing the parameters of echocardiographic RV functions. In the present study, both RV-MPI and RVFAC favorably reflected the hemodynamics in patients with CTEPH. Previously, Amano et al. reported that RV-MPI is a surrogate marker for the right ventricular ejection fraction (RVEF) assessed by MRI in patients with CTEPH [22]. RV-MPI is an index that combines RV systolic and diastolic function to evaluate RV function. Considering that the mean PAP was relatively high in our cohort (36.9 ± 12.0 mmHg), not only systolic function but also diastolic function would be impaired. Therefore, we speculated that RV-MPI showed a good correlation with hemodynamics in our cohort. As for exercise capacity, which was also important factor in previous report [23], some relationship was confirmed, although there was no statistically difference. RVFAC is known to reflect RV function; RVFAC has been shown to have a good correlation with RVEF measured by MRI and to be an independent predictor of mortality after pulmonary emboli [24]. RVFAC was reported to provide relevant clinical and prognostic information of pulmonary arterial hypertension when combined with the result obtained by CPET [25]. In accordance with these results, RVFAC correlated with hemodynamic status and exercise capacity the most, implying that RVFAC would be the best parameter among the four RV function parameters.

In contrast, TAPSE and *S*′ did not show strong correlations with hemodynamics and exercise capacity. Kind et al. reported that transverse measurement rather than longitudinal assessment, such as TAPSE and *S*′, reflects RVEF in pulmonary hypertension [26], In addition, TAPSE and *S*′ could be affected by RV enlargement and clockwise rotation of the apex of the heart [27]. Therefore, RV enlargement in patients with CTEPH might overestimate TAPSE and *S*′. But, actually TAPSE showed significant correlation with 6MWD (*r* = 0.340, *p* = 0.049) in our study (data not shown). There is also another report that resting TAPSE and *S*′ also showed moderate correlations with peakVO_2_ [28], and RV *S*′ < 10.5 cm/s raise suspicion for worse response to vasodilators in patients with CTD-PAH [29]. Although both TAPSE and *S*′ are used for the evaluation of the RV longitudinal movement, there are few reports clearly representing the difference between TAPSE and *S*′ in patients with CTEPH. Indeed, TAPSE is the index of distance of RV longitudinal movement, while *S*′ is that of speed of RV longitudinal movement. Considering relatively low correlation coefficient between TAPSE and *S*′ shown in Table 3 (*r* = 0.603), we finally proposed to evaluate comprehensive RV function by adding both TAPSE and *S*′ into RV dysfunction score in our study.

The 2015 ESC/ERS guidelines recommended the comprehensive prognostic evaluation and risk assessment for PAH patients since the single variable provides insufficient prognostic information [30]. However, the risk predictor in CTEPH patients was not shown previously as far as we know. Considering that the pathophysiology of CTEPH is caused not only by the obstruction of pulmonary artery by fibrotic transformation of pulmonary artery clots, but also by vascular remodeling in the microvasculature similar to PAH [31], a comprehensive risk assessment should also be done in patients with CTEPH at the time of initial risk assessment, treatment response assessment, and clinical worsening assessment [32]. Therefore, we compared the RV dysfunctional score with the variables recommended in the PAH guidelines for the evaluation of the CTEPH patients. The results showed that the RV dysfunction score showed good correlation with each important parameter of PAH. Thus, we concluded the RV dysfunction score was useful for evaluating the CTEPH prognosis.

The strength of the RV dysfunction score is that it can evaluate the patient’s status non-invasively and comprehensively. It could be useful not only for assessing RV function during the course of CTEPH treatment but also for predicting CTEPH prognosis.

Our study had several limitations. First, this study was single-center study and the sample size was relatively small. Because of few clinical events, we could not examine a relationship between the RV dysfunction score and prognosis in our cohort. Second, we have not determined whether this RV dysfunction score is suitable for patients with other types of pulmonary hypertension. Third, as intra-observer variability was not assessed, and the reproducibility of RV echocardiographic parameters could not be evaluated. Fourth, as we did not correct weighting, we could not prove each of the four RV echocardiographic parameters had equal value. Finally, we did not measure speckle-tracking strain, which has been used recently as a useful index for the assessment of RV function [33–35], RV dyssynchrony [36], and 3-dimensional assessment [37].

In conclusion, we proposed an RV dysfunction score using the four RV echocardiographic parameters (TAPSE < 16 mm, *S*′ < 10 cm/s, RVFAC < 35%, and RV-MPI > 0.4) in patients with CTEPH and demonstrated that the RV dysfunction score represents patients' characteristics on admission and hemodynamics. This RV dysfunction score could be a simple and useful scoring system providing a good estimation for hemodynamics when treating patients with CTEPH before catheterization.

## Electronic supplementary material

Below is the link to the electronic supplementary material.

Supplementary material 1 (DOCX 17 kb)
