# Supplementary material for: Usefulness of scoring right ventricular function for assessment of prognostic factors in patients with chronic thromboembolic pulmonary hypertension
Source: Heart Vessels. 2018 Apr 27;33(10):1220–8. doi: 10.1007/s00380-018-1168-7 (PMC6133067; doi:10.1007/s00380-018-1168-7)
Supplement: Supplementary file 1 — Supplementary material 1 (DOCX 17 kb) [file 380_2018_1168_MOESM1_ESM.docx]

**Supplementary Table 1.** Comparison between Riociguat (+) group and Riociguat (-) group.

|  | Riociguat (+) | Riociguat (-) |  |
| --- | --- | --- | --- |
|  | (n = 21) | (n = 14) | p value |
|  |  |  |  |
| **RV echo parameters** |  |  |  |
| TAPSE (mm) | 19.5 ± 3.8 | 17.6 ± 6.0 | 0.260 |
| S' (cm/sec) | 12.3 ± 3.1 | 11.4 ± 3.1 | 0.424 |
| RVFAC (%) | 32.1 ± 10.6 | 35.6 ± 17.9 | 0.474 |
| RV-MPI | 0.39 ± 0.13 | 0.41 ± 0.21 | 0.704 |
|  |  |  |  |
| **Hemodynamics** |  |  |  |
| mean PAP (mmHg) | 37.6 ± 11.5 | 36.6 ± 9.4 | 0.793 |
| CI (l/min/m^2^) | 2.5 ± 0.7 | 2.2 ± 0.7 | 0.267 |
| PVR (wood Unit) | 7.8 ± 4.9 | 8.7 ± 4.6 | 0.575 |
| RAP (mmHg) | 5.7 ± 3.0 | 6.5 ± 3.0 | 0.424 |
| SvO_2_ (%) | 63.2 ± 7.3 | 62.4 ± 11.1 | 0.787 |
|  |  |  |  |
| **Laboratory data** |  |  |  |
| BNP (pg/ml) | 116.2 ± 194.7 | 143.7 ± 175.0 | 0.674 |
|  |  |  |  |
| **Exercise capacity** |  |  |  |
| 6MWD (m) | 416.2 ± 106.5 | 370.3 ± 115.1 | 0.240 |
| peakVO_2_ (ml/min/kg) | 14.3 ± 3.3 | 13.2 ± 3.3 | 0.349 |
| VE/VCO_2_ | 42.2 ± 12.5 | 50.0 ± 16.0 | 0.120 |
|  |  |  |  |

Data are presented as mean ± standard deviation. TAPSE, tricuspid annular plane systolic excursion; S’, tissue doppler-derived tricuspid lateral annular systolic velocity; RVFAC, right ventricular fractional area change; RV-MPI, right ventricular myocardial performance index; BNP, brain natriuretic peptide; mean PAP, mean pulmonary artery pressure; CI, cardiac index; PVR, pulmonary vascular resistance; RAP, right atrial pressure; SvO_2_, mixed venous oxygen saturation; 6MWD, six minute walk distance; peakVO_2_, maximal oxygen consumption; VE/VCO_2_, minute ventilation/carbon dioxide production.
